# Validity of the Osteoarthritis Research Society International (OARSI) recommended performance-based tests of physical function in individuals with symptomatic Kellgren and Lawrence grade 0–2 knee osteoarthritis

**DOI:** 10.1186/s12891-022-06012-2

**Published:** 2022-12-01

**Authors:** Si-Huei Lee, Chi-Chun Kao, Huey-Wen Liang, Hung-Ta Wu

**Affiliations:** 1 Department of Physical Medicine and Rehabilitation, Taipei Veterans General Hospital, Taipei, Taiwan, Republic of China; 2 School of Medicine, National Yang Ming Chiao Tung University, Taipei, Taiwan, Republic of China; 3 Institute of Brain Science, National Yang Ming Chiao Tung University, Taipei, Taiwan, Republic of China; 4 Department of Physical Medicine and Rehabilitation, National Taiwan University Hospital and College of Medicine, No 7, Chong-Shan South Road, Taipei, 100 Taiwan, Republic of China; 5 Department of Physical Medicine and Rehabilitation, National Taiwan University Hospital Shin-Chu branch, Shin-Chu, Taiwan, Republic of China; 6 Department of Radiology, Taipei Veterans General Hospital, Taipei, Taiwan, Republic of China

**Keywords:** Knee, Osteoarthritis, Physical function, Outcome measures, Validity

## Abstract

**Background:**

Performance-based physical tests have been widely used as objective assessments for individuals with knee osteoarthritis (KOA), and the core set of tests recommended by the Osteoarthritis Research Society International (OARSI) aims to provide reliable, valid, feasible and standardized measures for clinical application. However, few studies have documented their validity in roentgenographically mild KOA. Our goal was to test the validity of five performance-based tests in symptomatic KOA patients with X-ray findings of Kellgren and Lawrence (K-L) grade 0–2.

**Methods:**

We recruited a convenience sample of thirty KOA patients from outpatient clinics and 30 age- and sex-matched asymptomatic controls from the community. They performed five OARSI-recommended physical tests and the KOA group answered the Western Ontario and McMaster Universities (WOMAC) Osteoarthritis Index. The tests included the 9-step stair-climbing test (9 s-SCT), timed up and go (TUG) test, 30-second chair-stand test (30sCST), 40-m fast walking-test (40MFPW) and 6-minute walking test (6MWT). The discriminant validity of these physical tests were assessed by comparisons between the KOA and control groups, receiver operating curve and multivariate logistic regression analysis. The convergent/divergent validity was assessed by correlation between the physical tests results and the three subscale scores of the WOMAC in the KOA group.

**Results:**

The KOA group had significantly worse performance than the control group. The percentage of difference was the largest in the 9 s-SCT (57.2%) and TUG tests (38.4%). Meanwhile, Cohen’s d was above 1.2 for the TUG test and 6MWT (1.2 ~ 2.0), and between 0.8 and 1.2 for the other tests. The areas under the curve to discriminate the two groups were mostly excellent to outstanding, except for the 30sCST. Convergent validity was documented with a moderate correlation between the 9 s-SCT and the physical function (WOMAC-PF) subscale scores (Spearman’s *ρ* = 0.60).

**Conclusions:**

The OARSI recommended core set was generally highly discriminative between people with K-L grade 0–2 KOA and their controls, but convergent/divergent validity was observed only in the 9 s-SCT. Further studies are required to evaluate the responsiveness of these tests and understand the discordance of physical performance and self-reported measures.

## Introduction

Knee osteoarthritis (KOA) is a common degenerative condition, with a prevalence of nearly 20% in American adults aged 45 years and older, and the trend is rising [1, 2]. It causes pain, swelling, limited joint range of motion and reduced leg muscle strength. Subsequently, patients have altered gaits and deteriorated ambulation, which leads to general functional decline and reduced quality of life [3]. KOA is among the most disabling conditions and is associated with limitations in walking and climbing stairs the most [4]. One study showed that KOA individuals had suboptimal physical activities compared with the general population, regardless of pain severity [5]. The adjusted percentage of disability attributable to OA was approximately 16%, and equal to or higher than nine other major conditions in four out of the seven functional items (walking, carrying, climbing stairs, and housekeeping) [6].

The impacts of KOA are multidimensional as described by the International Classification of Functioning Disability and Health (ICF), with high prevalence of the following secondary-level categories being reported: sensation of pain (96.3%) and mobility of joint (94.9%) for body function, lower extremity in body structure (93.2%), moving around (93.8%), changing basic body (90.1%), and walking (88.3%) for activity [7]. Therefore, a variety of tools have been proposed to characterize the impact of KOA for clinical practice, including patient-reported outcomes, clinical features, physical function outcomes and modifiable lifestyle-related outcomes [8]. Among the multiple assessment tools, patient-reported outcomes and objective measures of physical function are two major methods for assessing the domains of activities and participation. Several systematic reviews are available to discuss the application of outcome measurements for advanced or end-stage KOA, especially after knee arthroplasty [9, 10], but less for early-stage or mild KOA. It is noteworthy that early-stage or mild KOA may need separate measures for their wide range of ages and abilities concerning the potential floor and ceiling effects of outcome measurements [8]. Therefore, the selection of outcome measures in early KOA warrants further examination for both clinical practice and the research setting [8].

There are quite a few performance-based physical tests, and a standardized set facilitates efficient comparisons of treatment outcomes across studies. In response to these needs, the Osteoarthritis Research Society International (OARSI) recommends a set of performance-based tests of physical function as a core component of outcome measurement for individuals with hip or KOA or following joint replacement, based on available measurement-property evidence, feasibility of the tests, scoring methods and expert consensus [11]. The set of tests is considered representative of the typical activities relevant to the target population and includes five tests: the 30-second chair-stand test (30sCST), 40-m fast-paced walking test (40MFPW), stair-climbing test (SCT), timed up and go (TUG) test and 6-minute walking test (6MWT), with the first three tests as a minimal core set. Previous studies have documented their reliability among individuals with knee and/or hip OA [12], but the validity of these tests has not yet been universally agreed upon [13, 14]. In addition, the physical tests in the OARSI-recommended set are established mostly based on moderate-to-severe or end-stage OA and cannot be assumed to have adequate psychometric performance when applied in early OA [11]. The 10s-SCT and 30sCST had poor construct validity and responsiveness in the assessment of function among KOA patients pending for total knee arthroplasty (TKA) [13]. Meanwhile, the TUG is considered a reliable test with adequate minimal detectable change for clinical use in individuals with K-L grade I to III, but excluded from the OARSI recommended minimum core set [15]. Therefore, there is a need to test the validity of the OARSI-recommended physical tests in mild KOA patients.

Our goal was to establish the validity of the OARSI recommended core set for patients with roentgenographically mild KOA. Discriminant validity was assessed by comparing the performance-based physical performance between the KOA group and their healthy controls with no knee pain. Grade II was selected as a cutoff grading to exclude patients with definite joint space narrowing. We also tested convergent/divergent validity by comparing physical performance and a self-reported outcome measure. We hypothesized that physical performance would be worse in the KOA group than in the control group. Additionally, we hypothesized that physical performance would have moderate correlation with self-reported activity limitations but a low correlation with symptoms (pain and stiffness) in the KOA group, since the performance-based measure and self-reported symptoms captured a different construct of function.

## Method

### Study design and the participants

This was a case-control study. A convenience sample of participants was recruited from the Physical Medicine and Rehabilitation (PMR) outpatient clinics of the institutes involved in the study. To be eligible, participants were required to be: (1) aged more than 50 years; (2) diagnosed with unilateral or bilateral KOA according to the criteria of clinical and radiographic findings by the American College of Rheumatology (ACR), i.e., knee pain and at least one of the following symptoms: age more than 50 years, stiffness less than 30 minutes, crepitus, and osteophytes [16]; (3) receiving nonsurgical treatment for KOA in the PMR outpatient clinics of the study institutes in the past 3 months; (4) roentgenography of KL Grading Scale grade II or less [17]; and (5) able to independently ambulate without any walking aids in the community. The exclusion criteria were any history of other neuromuscular disorders of the lower limbs, visual deficits or cardiopulmonary disease that may interfere with walking and balance, and being unable to read or follow instructions. The KL grading was interpreted by one author (HTW) based on recent weight-bearing, anterior-posterior X-rays of the tibiofemoral joint for both knees without knowledge of the clinical conditions. An age- and sex-matched sample was recruited from the community. They could walk normally without a device, reported no knee pain in the past year and were not diagnosed with KOA. The exclusion criteria were the same as those for the KOA group.

Sample size was estimated based on a *t* test (the difference between two independent means), with the following factors: one-tailed, α error probability = 0.05, β error probability = 0.2 (i.e., power 1 - β =0.8 or 80%), and a moderate effect size (ES) of 0.66 [18]. This required 30 participants in each group. The study was approved by the research ethics committee of the National Taiwan University Hospital (approval no: 20180094RINB, date: 3/21/2019) and the Taipei Veterans Hospital (approval number: 2019–01-007A, date: 01/07/2019) and was in accordance with the Helsinki Declaration of 1975, as revised in 2000. All participants provided written informed consent before participation.

### Procedures

All the participants completed a questionnaire to provide basic characteristics, such as age, sex, body height, body weight, and exercise habits. Only the KOA group answered the Western Ontario and McMaster Universities Osteoarthritis Index (WOMAC) [19], which contains 24 items in three subscales measuring pain (WOMAC-P; 5 items), stiffness (WOMAC-S; 2 items), and physical function (WOMAC-PF; 17 items). The participants rated the items on a 5-point Likert scale (none, mild, moderate, severe, and extreme), and a summary score was calculated for the pain, stiffness, and physical function subscales, with maximum scores of 20, 8 and 68 respectively.

### Performance-based tests

Five performance-based physical tests were conducted according to the recommendation of the OARSI for the setup, procedures, verbal instructions and scoring [20]. The time was measured on a stopwatch to the nearest one-hundredth of a second, and the distance was measured to the nearest centimeter. The participants completed the five performance tests in the following order and 3-minutes were allowed between each test:

1. 40MFPW [21]: The participants walked as quickly and safely as possible on a 10-m walkway, turned around a cone placed 2 m beyond each end of the walkway and returned for a total distance of 40 m. None of the participants used a walking aid, and the time to complete the task was recorded. The intrarater and interrater intraclass correlation coefficient (ICC) were 0.92 and 0.96, respectively, in individuals with knee and hip OA [12]
2. TUG test [22]: The participants stood up from an armed chair, walked at a safe and comfortable pace to a line 3 m away, crossed the line, turned, and returned to a sitting position in the chair. None of the participants used a walking aid and the time to complete the task was recorded. The intrarater and interrater reliability (ICC) were 0.97 and 0.96, respectively, in individuals with doubtful to moderate KOA [15].
3. 30sCST [23]: The participants stood up completely from a sitting position from an unarmed and straight back chair (seat height: 45 cm); and then completely back down until they were completely on the seat. The maximum number of chair-stand repetitions completed in a 30-second period was recorded. The participants had a practice of two slow-paced repetitions before formal testing to ensure understanding. The test-retest ICC was 0.84 for men and 0.92 for women in community-dwelling older adults [23].
4. 6MWT [24, 25]: For 6 minutes, the participants walked back and forth as far a distance as possible on a 40-m unobstructed walkway with 2 cones at each end. Standardized encouragement was provided at 60-second intervals [25]. The intrarater and interrater reliability (ICC) were 0.93 and 0.94, respectively, in individuals with knee and hip OA [12].
5. 9-step SCT (9 s-SCT) [26]: The participants ascended and descended nine stairs (step height, 20 cm) as quickly as possible but in a safe manner. A handrail was available, but none of them used the handrail or walking aids. The time for the participants to complete the ascending and descending tasks were recorded. The test had a very high reliability (ICC = 0.98) among the patients after TKA [26].

### Data analysis

All the data were checked for normality with the Shapiro-Wilk test and descriptive analyses are presented as the mean and standard deviation or median and interquartile range as appropriate. Cohen’s d was used to evaluate the ES of these functional tasks in the KOA or control groups [27], with d = 0.2, 0.5 and 0.8 indicative of a small, medium and large ES, respectively [28]. The differences in demographic data and outcome variables were compared between the KOA and control groups with either independent *t* tests for parametric data or Mann-Whitney U tests for nonparametric data. We also performed a multivariate logistic regression using the grouping as the dependent variable and the results of the five performance tests as independent variables. Age and body height were adjusted to control confounding effects on performance. The discriminative power of these functional tests for the two groups was evaluated by receiver operating curve (ROC) analysis, with an area under the curve (AUC) of 0.9 and higher considered outstanding discrimination, 0.8 to 0.9 considered excellent discrimination and 0.7 to 0.8 considered acceptable discrimination [29]. The convergent/divergent validity was assessed with correlation analysis between each performance test result and the WOMAC Index subscale scores. Spearman’s *ρ* or Pearson’s correlation coefficient was computed depending on the distribution of the data. The size of the correlation coefficient was interpreted as very high (0.90), high (0.7 to 0.9), moderate (0.5 to 0.7) or low (0.3 to 0.5) [30]. SPSS (version 21, SPSS Chicago, IL USA) was used to perform statistical analyses.

## Results

Thirty KOA and 30 control subjects completed all five functional performance tasks. These two groups had similar ages, sex ratios and body mass index (BMI) (Table 1). Approximately 77% of the KOA group had a pain duration of more than 1 year and were receiving at least one kind of treatment. All knee X-rays were graded as KL classification II or less. The subscale scores were on average 8.8 ± 3.8 out of 20 for the WOMAC-P, 3.4 ± 1.7 out of 8 for the WOMAC-S, and 27.8 ± 13.2 out of 68 for the WOMAC-PF.

**Table 1:** Demographic data of all subjects and clinical characteristics of 30 osteoarthritic subjects

	Osteoarthritic group (*N* = 30)	Control group (*N* = 30)	^a^*p* vale
Female, n (%)	27 (90%)	27 (90%)	1.000
Age (years)	63.2 ± 6.5	62.9 ± 6.6	0.875
Body height (cm)	157.1 ± 6.4	157.3 ± 5.3	0.896
Body weight (kg)	59.5 ± 14.1	54.9 ± 8.2	0.124
Body mass index (kg/m^2^)	24.1 ± 5.1	22.1 ± 2.7	0.075
Overweight, n (%)	11 (36.7%)	4 (13.3%)	0.072
Employed	9 (30.0%)	6 (20.0%)	0.371
Sport habits, n (%)			0.451
Nil	9 (30.0%)	5 (16.7%)	
Irregular	5 (16.7%)	7 (23.3%)	
Regular	16 (53.3%)	18 (60.0%)	
Overall pain duration > 1 year	23 (76.7%)	–	
Treatment in the past 3 months, n (%)			
Medication	11 (36.7%)	–	
Injection	13 (43.3%)	–	
Physical therapy	14 (46.7%)	–	
Others	5 (16.7%)	–	
Current knee pain under treatment, n (%)			
Bilateral	8 (36.7%)	–	
Unilateral	22 (30.0%)	–	
WOMAC, median (interquartile range)	34.0 (15.0)	–	
Pain subscale	8.5 (5.0)	–	
Stiffness subscale	3.0 (3.0)	–	
Physical function subscale	23.0 (9.3)	–	
Kellgren and Lawrence classification for both knees, n (%)			
Zero	29 (48.3%)	–	
I	16 (26.7%)	–	
II	15 (25%)	–	

*WOMAC* Western Ontario and McMaster Universities Osteoarthritis Index

^a^Compared with independent *t* test for continuous data and Fisher’s Exact test or Chi square test for categorical data

All the physical test results in the KOA group and parts of the test results in the control group violated a normal distribution. The KOA group generally had significantly worse performance in all functional tests based on the Mann-Whitney U tests (Table 2). The difference was the largest in the 9 s-SCT (57.2%) and TUG tests (38.4%). Meanwhile, Cohen’s d was above 1.2 for the TUG test and 6MWT (1.2 ~ 2.0), and between 0.8 and 1.2 for the other tests.

**Table 2:** Physical performance results in the osteoarthritic and control groups are presented as the means±standard deviations and medians and interquartile ranges in parentheses

	Osteoarthritic group (*N* = 30)	Control group (*N* = 30)	Difference %	Cohen’s’ d	95% CI of effect size	^a^*p* value
40-m fast-paced walking (seconds)	32.5 ± 12.0 (30.5, 7.2)	24.6 ± 3.0 (24.0, 4.3)	27.7%	−0.90	− 1.43 ~ − 0.37	< 0.001
9-step stair-climbing test (seconds)	24.1 ± 12.8 (21.1, 9.3)	13.3 ± 1.9 (13.3, 2.4)	57.2%	−1.17	−1.72 ~ −0.62	< 0.001
30s chair-stand test (counts)	10.6 ± 3.5 (9.8, 6.0)	14.7 ± 5.2 (13.0, 4.6)	−31.7%	0.90	0.37 ~ 1.43	< 0.001
6-minute walking test (meters)	392.8 ± 73.0 (388.3, 74.9)	470.1 ± 45.0 (467.5, 47.6)	−17.5%	1.23	0.68 ~ 1.78	< 0.001
Timed up and go (seconds)	10.8 ± 3.5 (10.5, 2.8)	7.3 ± 0.9 (7.2, 1.6))	38.4%	−1.36	−1.92 ~ −0.80	< 0.001

*CI* Confidence interval

^a^comparison with Mann-Whitney U test

A multivariate logistic regression analysis was performed to adjust the confounding demographic factors (age and body height) on the physical performance results to classify the KOA group and controls (Table 3). All physical function test results were associated with being in the KOA group after other factors were held constant.

**Table 3:** Physical performance tests were used to discriminating osteoarthritic and normal participants by receiver operating curve analysis

Tests	AUC	95% CI	*p* value	Cut-point	Sensitivity	Specificity
40-m fast-paced walking	0.87	0.78 ~ 0.96	< 0.001	26.8	0.77	0.80
9-step stair-climbing test	0.94	0.87 ~ 1.00	< 0.001	15.5	0.90	0.93
30s chair stand test	0.76	0.63 ~ 0.88	0.001	9.8	0.50	1.00
6-minute walking test	0.84	0.73 ~ 0.97	< 0.001	421.5	0.73	0.90
Timed up and go	0.91	0.83 ~ 0.99	< 0.001	8.5	0.83	0.90

*AUC* Area under curve, *CI* Confidence interval

**Fig. 1:**
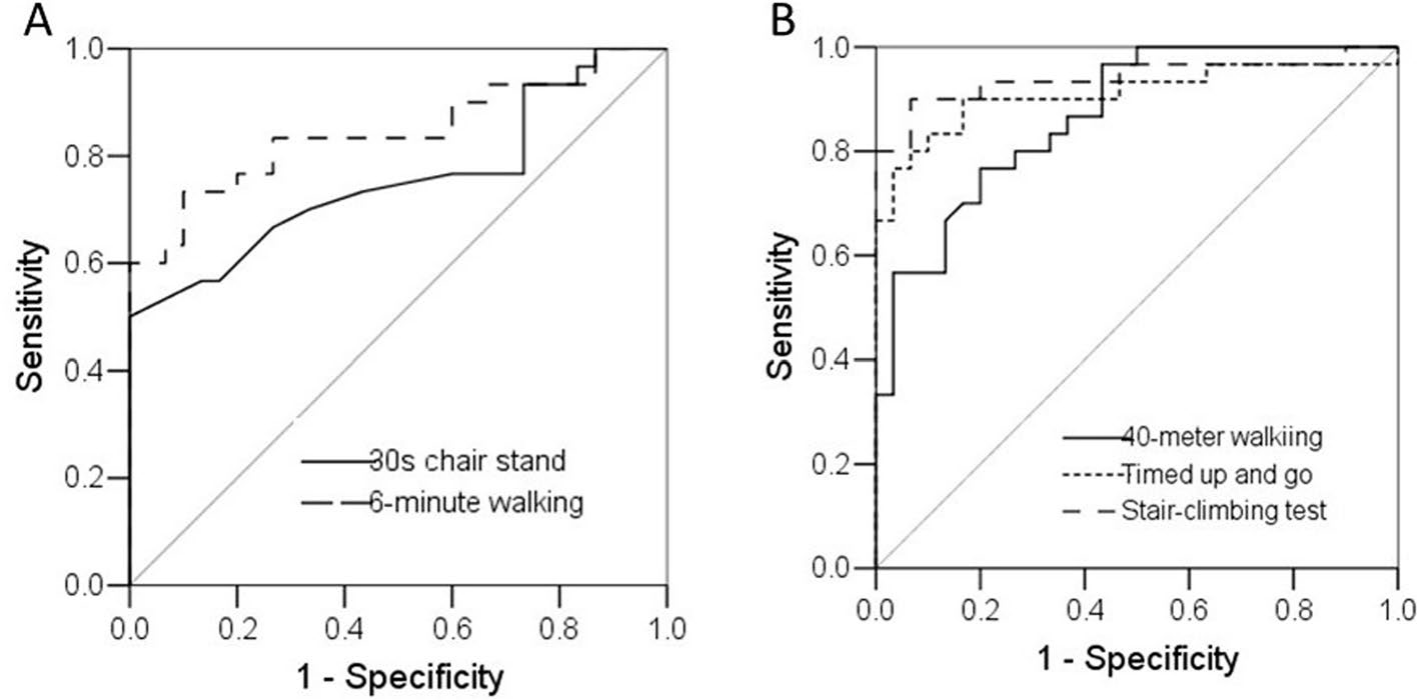
The results of receiver operating curve analysis for fiver performance tests to discriminate osteoarthritic and normal participants

The AUCs for the five performance-tests to discriminate the KOA and control groups were mostly excellent to outstanding (0.84 to 0.94), except for the 30sCST (0.76) (Table 4, Fig. 1). The 9 s-SCT had the highest AUC, with a sensitivity of 0.9 and a specificity of 0.93. Meanwhile, the 30sCST had the highest specificity (1.0), but lower sensitivity (0.5).

**Table 4:** Results of multivariate logistic regression with each performance test as independent variables and adjusted for age and body height to predict osteoarthritis group

	Odds ratio (95% confidence interval)	*p* value
40-m fast-paced walking	1.82 (1.33 ~ 2.48)	< 0.001
9-step stair-climbing test	2.56 (1.54 ~ 6.26)	< 0.001
30s chair stand test	0.76 (0.63 ~ 0.91)	0.004
6-minute walking test	0.97 (0.96 ~ 0.99)	< 0.001
Timed up and go	3.60 (1.92 ~ 6.78)	< 0.001

The correlations between the results in each performance test and the subscale and WOMAC-T were calculated with Spearman *ρ* because of violations of a normal distribution. Only the 40MFPW and 9 s-SCT had low to moderate associations with the WOMAC-PF subscale and WOMAC-T scores (ρ = 0.42–0.60) (Table 5). The TUG, 30sCST and 6MWT scores had mostly no correlations with the WOMAC scores.

**Table 5:** Spearman’s ρ correlation coefficient and *p* value between the performance tests and WOMAC scores

WOMAC	40-m fast-paced walking	9-step stair-climbing test	30s chair stand test	6-minute walking test	Timed up and go
Pain subscale	0.39 (0.03)	0.36 (0.51)	0.04 (0.82)	−0.13 (0.51)	0.14 (0.47)
Stiffness subscale	0.30 (0.11)	0.27 (0.23)	0.32 (0.08)	−0.06 (0.77)	−0.10 (0.61)
Physical function subscale	0.42 (0.02)	0.60 (0.001)	−0.04 (0.84)	−0.36 (0.049)	0.13 (0.50)
Total score	0.54 (0.002)	0.51 (0.004)	−0.04 (0.83)	−0.29 (0.12)	0.14 (0.46)

*WOMAC* Western Ontario and McMaster Universities Osteoarthritis Index

## Discussion

We tested the validity of a set of performance-based tests recommended by the OARSI for individuals with mild KOA, who had no to mild changes on X-ray (KL classification of II or less). The discriminative validity was supported with the KOA group having significantly worse physical test results than the control group, even after adjustment for demographic characteristics. Based on ROC analysis, the 9 s-SCT had the highest sensitivity and the 30sCST had the highest specificity. In addition, convergent/divergent validity was observed only for the 9 s-SCT, which had moderate correlations with WOMAC-PF scores and low or no correlation with WOMAC-P and WOMAC-S scores. Our study was different from previous studies, as it included a complete set of OARSI-recommended performance tests and targeted KOA patients with preradiographic to mild changes but not patients in advanced or peri-TKA stages. The clinical significance warrants further exploration for further application.

Our inclusion criteria for KOA were based on the clinical criteria of the ACR, which include knee pain and at least one of the following symptoms or signs: age of 50 years or older, stiffness lasting less than 30 minutes, crepitus and bone osteophytes on X-ray. These criteria have a slightly lower sensitivity (91%) but higher specificity (86%) than clinical criteria alone or the combination of clinical and laboratory criteria [16]. Although more than two-thirds of the participants reported having knee pain for more than 1 year, just over half of them had roentgenographic changes. A limited number of studies have revealed possible activity limitations even in this early stage [4, 31]. This set of performance-based tests was proposed by the OARSI through an extensive literature review and consensus from 138 experienced experts from 16 countries [11]. These tests generally have sufficient to optimal within-rater and interrater reliability [12, 32], but their validity has not been universally agreed upon [33]. Moreover, available data from the recommended tests, at best, support their use in middle-aged and older people with moderate-to-severe or end-stage OA, and their generalizability to people with very early disease has yet to be confirmed.

We first tested the discriminant validity by examining the ability of these tests to detect known-group differences: in this study, differences between mild KOA patients and age- and sex-matched healthy controls were examined. The results supported the hypothesis that this group of KOA individuals with preroentgenographic or early-stage changes had significantly worse performance scores than the control group in all tests. Cohen’s d was larger than 1.0 for the TUG test, 9 s-SCT, and 6MWT, and was 0.9 to 1.0 for the 40MFPW and 30sCST. This trend is similar to previous studies showing that the complete set of performance-based physical tests or parts of this set were adequate to discriminate healthy and moderate to advanced OA [13, 14, 34], with a large ES for the 10s-SCT (Cohen’s d = 1.3), TUG (Cohen’s d = 0.9) and 6MWT (Cohen’s d = 0.9), and a moderate ES for the 30sCST (Cohen’s d = 0.5) [34]. The discriminant validity remained after we controlled for age and body height with multivariate logistic regression analysis [35]. With the ROC analysis, the AUCs of the five physical function tests were all above 0.75, with the 9 s-SCT and TUG test having the highest AUCs. Collectively, we suggest that the 9 s-SCT and the TUG test have the highest levels of discriminative validity among these five tests. The early influence on stair activities in KOA patients has been documented through functional tests and self-reported outcomes. For example, the first patient-reported activity in the WOMAC questionnaire that is associated with knee pain is “using stairs” according to Rasch modeling [4]. The 9 s-SCT time, especially the ascending time value, is useful for identifying early KOA patients (K-L grade I) [31], and stair-climbing ability is more affected by pain catastrophizing than the ability to stand from a seated posture and walk in KOA patients [36]. However, the use of the 9 s-SCT may have limited feasibility in clinical practice. Comparatively, the TUG test represents abilities related to ambulatory transitions and evaluates leg strength and balance. One study regarded the TUG test as a reliable test with an adequate minimal detectable change in individuals with low to moderate knee OA (grades 1 to 3) [15]. This test ranked the highest in terms of clinical feasibility but was less preferred than the 30sCST among the sit-to-stand tests in the consensus process to select the OARSI recommended set [11]. Ultimately, the TUG test is not included in the minimum core tests for overlap of the activity themes with the 30sCST. However, it seemed to be a more sensitive tool than the 30sCST among mild KOA patients in terms of discriminative ability.

According to our results, the hypothesis of moderate correlation between functional performance results and self-reported activity limitation (WOMAC-PF scores) was supported only with the 9 s-SCT. Moreover, mostly low or no correlation was observed between the functional performance results and self-reported pain and stiffness, supporting divergent validity. The discordance or low correlation between the performance tests and self-reported activity limitation has been reported previously. For example, poor construct validity and responsiveness were reported for the sit-to-stand movement, walking short distances and stair negotiation among KOA patients with indication for TKA [13]. Another study showed a low correlation (*r* = 0.33) between the TUG and WOMAC-PF normalized scores in KOA patients prior to TKA. A subgroup analysis showed that young individuals tend to have higher (i.e., worse) self-reported scores than performance-based scores [37]. One possible explanation is disablement process theory [38], which suggests that the individuals’ expectations of their abilities are associated with their responses to their disablement experiences during daily activities. For example, younger KOA participants report more distress and frustration managing the disease and a greater impact of their health on work, leisure, social activities, and relationships than older controls [39]. In addition, sex, obesity and pain catastrophizing, and number of symptomatic joints were associated with discordance [37].

Discordance between physical functional performance and self-reported activity limitation is considered a rationale to use both self-reported outcome measures and performance tests as complimentary assessments [40–42]. Moreover, discordance raises another issue for selecting measures to assess the convergent validity of these physical performance tests. In addition to self-reported activity limitations, several other criteria have been proven to have only a weak correlation with physical performance, such as knee extensor strength, KL staging and quality of life [13, 43, 44]. Therefore, construct validity needs to be evaluated with other psychometric properties, such as responsiveness, to determine the best outcome measures in this group of KOA patients.

## Limitations

Three limitations should be addressed. First, up to 90% of the participants were women, which raises doubts regarding generalizability to men. This could be related to the use of a convenience sample, but also probably reflects the increased risk of KOA in females [45]. Second, the functioning of an individual with KOA, according to the ICF model, is the collaborative interaction among a person’s health condition, environmental factors and personal factors [46], which were not measured in detail in the current study. Previous studies showed that other psychosocial or demographic data were major determinants of physical performance or self-reported outcomes [47]. These data should be collected with a larger sample size for an in-depth analysis. Finally, the control group was based on medical history, and no X-rays of their knees were taken. Therefore, we could not rule out the presence of roentgengraphic findings. The discriminative physical performance could be attributed to the pain, rather than the roentgengraphic change or loss of strength related to disuse or chronic pain.

## Conclusion

The clinical course of KOA involves a slow progression, and the long course offers a wide window of opportunity to alter its course and identify effective approaches for early identification and management [48]. Therefore, choosing reliable and valid outcome measures for mild or early KOA is crucial. The OARSI recommended performance tests can discriminate mild KOA patients and controls. The 6MWT, 9 s-SCT and TUG test are the preferred options due to their excellent discriminative ability and large ES. Notably, these three tests are different from the minimal core set recommended by the OARSI. Convergent/divergent validity with self-reported activity limitation/symptoms was observed only in the 9 s-SCT. Nonetheless, the feasibility of using the 9 s-SCT may be limited in outpatient clinics and the TUG test can be used as an alternative test for individuals with mild KOA. Responsiveness, an important indicator of construct validity, should be tested in future studies to help select outcome measures for this group of patients.

## Data Availability

The datasets used and/or analyzed during the current study The datasets generated and/or analyzed during the current study are not publicly available due to the restriction under the institutional ethical committee‘s policy, but may be available from the corresponding author on reasonable request and with permission of the ethical committee.

## Abbreviations

30sCST: 30-second chair-stand test
9 s-SCT: 9-step stair-climbing test
40MFPW: 40-m fast-paced walking test
6MWT: 6-minute walking test
ACR: American College of Rheumatology
AUC: Area under the curve
BMI: Body mass index
ES: Effect size
ICF: International Classification of Functioning Disability and Health (ICF
K-L: Kellgren and Lawrence
KOA: Knee osteoarthritis
OA: Osteoarthritis
OARSI: Osteoarthritis Research Society International
PMR: Physical Medicine and Rehabilitation
ROC: Receiver operating characteristic
SCT: Stair-climbing test
TKA: Total knee arthroplasty
TUG: Timed up and go
WOMAC: Western Ontario and McMaster Universities Osteoarthritis Index
WOMAC-P: Pain subscale of the Western Ontario and McMaster Universities Osteoarthritis Index
WOMAC-PF: Physical function subscale of the Western Ontario and McMaster Universities Osteoarthritis Index
WOMAC-S: Stiffness subscale of the Western Ontario and McMaster Universities Osteoarthritis Index
WOMAC-T: Total score of the Western Ontario and McMaster Universities Osteoarthritis Index
